# A Rare but Morbid Occurrence: Development of Glioblastoma Multiforme During Tumor Necrosis Factor Inhibitor Therapy

**DOI:** 10.7759/cureus.25027

**Published:** 2022-05-15

**Authors:** Laura S Kraemer, Ross J Humes, Azfar S Syed, Adam M Tritsch

**Affiliations:** 1 Internal Medicine, Naval Medical Center San Diego, San Diego, USA; 2 Gastroenterology, Dwight D. Eisenhower Army Medical Center, Augusta, USA; 3 Gastroenterology, Walter Reed National Military Medical Center, Bethesda, USA

**Keywords:** ibd management, drug induced, biologics, glioblastoma multiforme, tnf inhibitors

## Abstract

The use of biologic therapies continues to become more prevalent in the treatment of inflammatory bowel disease, particularly for more severe disease. Although generally safe and effective, specific biologic classes such as tumor necrosis factor inhibitor (anti-TNF) medications are known to increase the risk of certain cancers. Glioblastoma multiforme (GBM) is an aggressive brain tumor which tends to arise sporadically but may be associated with anti-TNF therapies. Here, we present a case of a 69-year-old male with Crohn’s disease who developed GBM while on adalimumab therapy. This case report highlights the potential rare association between GBM and anti-TNF therapy and further discusses the difficulty of managing active Crohn’s disease with concomitant GBM, specifically the difficulty encountered in managing a disease flare.

## Introduction

Biologics have rapidly gained favor in controlling inflammatory bowel disease (IBD), particularly moderate to severe Crohn’s disease (CD) due to their efficacy and favorable side effect profile when compared to corticosteroids and immunomodulators. In less than a decade, a large proportion of CD patients have received biologic therapy with a reported increase from 21.8% in 2007 to 43.8% in 2015 [1]. Additionally, 15% of CD patients in a population-based study were treated with a biologic therapy within five years from initial diagnosis [2-3]. Considering these statistics, it is likely that the percentage of CD patients on biologics will only continue to increase over time [2].

Commonly prescribed tumor necrosis factor inhibitors (anti-TNF) currently available for the treatment of CD in the United States include infliximab (Remicade®), adalimumab (Humira®), and certolizumab (Cimzia®) [4-5]. Although anti-TNF therapies are generally safe, well-tolerated medications, severe side effects can occur including infusion reactions, infections, and malignancies [2,6,7]. Lymphoma and non-melanoma skin cancer are malignancies typically associated with anti-TNF therapy with other types occurring at a similar rate as the general population, according to a very large safety review done on patients treated with adalimumab [8]. While inhibition of tumor necrosis factor (TNF) can lead to CD remission through the dampening of inflammatory responses such as the inappropriate differentiation of monocytes to macrophages, neutrophil recruitment, and granuloma formation, long term TNF suppression is associated with cell-mediated immunosuppression leading to reduced cellular apoptosis, promotion of tumorigenesis, and increased intracellular pathogen replication. Thus, anti-TNF therapies may increase the rate of tumor development, growth, and malignant potential [2,5].

Currently, the only known environmental factor linked to primary brain cancer is ionizing radiation [9]. Recently, there has been increased investigation between the relationship of anti-TNF therapies and glioblastoma multiforme (GBM), the most aggressive type of primary brain cancer with a reported median survival time of 15-20.9 months from the time of diagnosis [5,9]. It has been suggested that infliximab and adalimumab have a stronger association with GBM compared to other anti-TNF agents [5]. Here we outline a rare case of GBM in an individual whose CD was well controlled anti-TNF therapy.

## Case presentation

**Figure 1:**
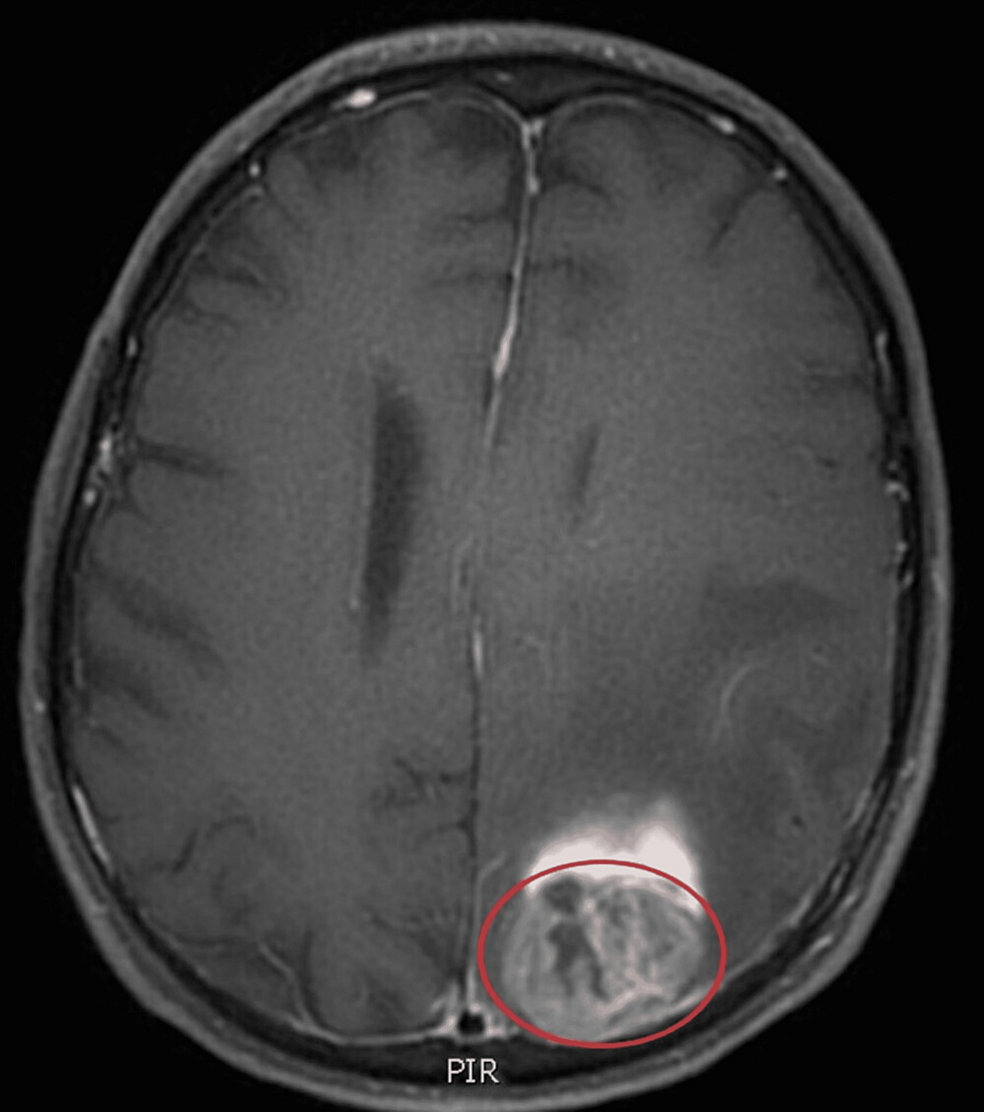
MRI Brain with contrast enhancement T1 image showing central clearing, heterogeneous contrast enhancement with rim enhancement. Consistent with glioblastoma multiforme.

Our case pertains to a 69-year-old male with fibrostenotic ileal CD first diagnosed in 1995, status post ileocecectomy for small bowel obstruction (SBO) in 1997, and current therapy with weekly 40mg adalimumab since 2014. His other medical problems include cervicalgia which required surgery in the past, and a history of skin cancer (melanoma/basal cell carcinoma). Outside of a handful of minor flares, his symptoms have been well controlled with adalimumab. In September 2020, he presented with symptoms of worsening right-sided hemiparesis and aphasia. Imaging obtained at the time revealed a left parietal mass which was confirmed as a GBM on MRI (Figure 1). Since our patient had no other risk factor for GBM, adalimumab was discontinued; and he underwent extensive treatment consisting of surgery, radiation therapy, and palliative Temozolomide with tumor treatment field therapy utilizing the Optune® device.

His CD symptoms returned approximately two months after discontinuation of adalimumab and consisted of increased stool frequency, fecal urgency, and right lower quadrant abdominal pain. Restarting adalimumab was considered despite the risk of tumor progression. His inflammatory markers (CRP, fecal calprotectin) were normal alongside a negative infectious workup and he had no nocturnal bowel movements, which he had during his previous flares. A short course of corticosteroids was given alongside of a psyllium fiber supplement. Due to complete symptomatic resolution within two weeks of initiating the aforementioned therapy, endoscopic evaluation was not completed and corticosteroids were discontinued. Fiber supplement was continued and it is thought his flare symptoms were due to concomitant irritable bowel syndrome with predominant diarrhea (IBS-D). He is currently not on any maintenance therapy for his CD, with symptoms still in remission.

## Discussion

Anti-TNF therapies, such as adalimumab, are growing in use due to their effectiveness in treating CD. It is well reported in the literature that TNF is essential in the regulation of the immune system including deterring tumorigenesis. In vivo studies have shown inhibition of glioma cell growth with cellular transfection with the TNF-a gene suggesting TNF may play an essential role in reducing glioma progression [5,10-13]. This results in a management conundrum, especially after the development of a tumor and the need for continued treatment of CD.

Despite a plethora of pharmacologic therapies available to include anti-TNF therapies, there are a substantial number of patients who show poor clinical response and never fully reach remission of their IBD symptoms [14]. Therefore, discontinuing an effective therapy when adverse effects develop becomes a difficult decision. There is limited knowledge on the risk of tumor progression while receiving concomitant GBM and anti-TNF therapy, with a theoretical risk of tumor progression with TNF suppression. Hence, cessation of anti-TNF therapy is ideal in the appropriate context.

Fortunately for our patient, his symptoms were found to be related to overlying IBS-D. His treatment options would have been limited in the case of an active CD flare. Corticosteroids are a temporary option that generally work within days to achieve a clinical response but their use should be limited due to numerous side effects. Other treatment options include immunomodulatory therapy such as methotrexate and 6-MP which can take weeks to take effect. Newer biologic therapies that do not work by the anti-TNF mechanism such as vedolizumab and ustekinumab are a consideration, but carry a theoretical risk of malignancy as well. Specifically, long-term data on the development of brain tumors is lacking with these agents and the frequency is not yet known. Lastly, these medications would also take weeks to take effect which limits their utility in an acute flare.

If uncontrolled CD flare symptoms were to develop, it can result in complications and symptoms that may result in early termination of chemotherapy. GBM has a poor prognosis with a 2%, two-year survival rate for patients ≥ 65 years old [5]. Decreasing this short life expectancy and further negatively impacting the remaining quality of life is not desirable. Ultimately, more research is needed to study the optimal treatment strategies in patients who have to discontinue effective biologic therapies in the setting of active disease, and the association of anti-TNF therapy with GBM.

## Conclusions

Our case highlights the rare development of GBM in an individual on anti-TNF therapy, which presents with both challenges in management and portends a poor overall prognosis; a potential association patients should be educated on. With their growing use in the IBD population, more patients on these therapies will be encountered by clinicians in all specialties. Clinicians should become very familiar with these therapies and maintain a high index of suspicion for their potential association with rare malignancies.
